# Looking Beyond the Terrestrial: The Potential of Seaweed Derived Bioactives to Treat Non-Communicable Diseases

**DOI:** 10.3390/md14030060

**Published:** 2016-03-18

**Authors:** Kenneth G. Collins, Gerald F. Fitzgerald, Catherine Stanton, R. Paul Ross

**Affiliations:** 1 kenneth.collins@teagasc.iep.ross@ucc.ie; 2 g.fitzgerald@ucc.ie; 3

**Keywords:** seaweed, bioactives, non-communicable disease, cancer, diabetes, cardiovascular disease, fucoidan, short-chain fatty acids, prebiotics

## Abstract

Seaweeds are a large and diverse group of marine organisms that are commonly found in the maritime regions of the world. They are an excellent source of biologically active secondary metabolites and have been shown to exhibit a wide range of therapeutic properties, including anti-cancer, anti-oxidant, anti-inflammatory and anti-diabetic activities. Several Asian cultures have a strong tradition of using different varieties of seaweed extensively in cooking as well as in herbal medicines preparations. As such, seaweeds have been used to treat a wide variety of health conditions such as cancer, digestive problems, and renal disorders. Today, increasing numbers of people are adopting a “westernised lifestyle” characterised by low levels of physical exercise and excessive calorific and saturated fat intake. This has led to an increase in numbers of chronic Non-communicable diseases (NCDs) such as cancer, cardiovascular disease, and *diabetes mellitus*, being reported. Recently, NCDs have replaced communicable infectious diseases as the number one cause of human mortality. Current medical treatments for NCDs rely mainly on drugs that have been obtained from the terrestrial regions of the world, with the oceans and seas remaining largely an untapped reservoir for exploration. This review focuses on the potential of using seaweed derived bioactives including polysaccharides, antioxidants and fatty acids, amongst others, to treat chronic NCDs such as cancer, cardiovascular disease and *diabetes mellitus*.

## 1. Introduction

**Figure 1:**
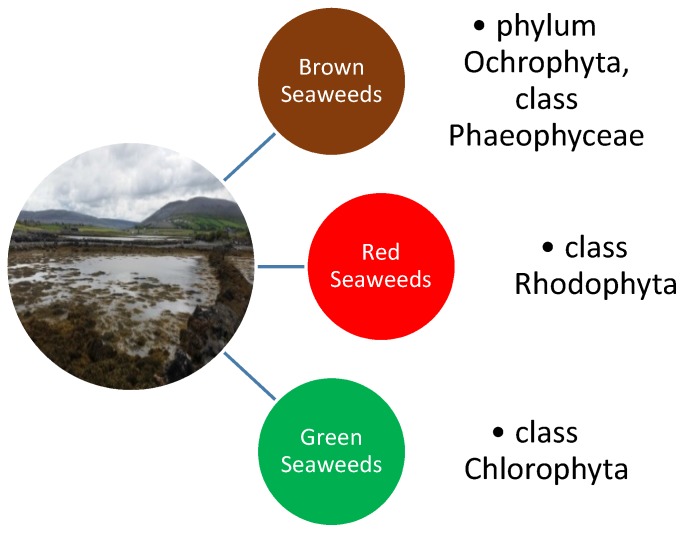
Seaweeds are divided into three main groupings based largely on their pigmentation. The groupings are brown seaweeds, the red seaweeds and the green seaweeds.

Seaweeds are an extensive group of autotrophic organisms that have a long fossil history. They are globally distributed and can be found in every climatic zone ranging from the tropical warm waters to freezing cold Polar Regions [1]. At present, more than 10,000 different species of seaweed are known [2]. The traditional division of the various seaweed species is one based largely on differences in pigmentations. The three main groupings are; the Brown seaweeds (phylum Ochrophyta, class Phaeophyceae), the Red seaweed (phylum Rhodophyta) and the Green seaweeds (phylum Chlorophyta) (Figure 1) [3]. Seaweeds have been extensively used by mankind since the beginnings of recorded history in a wide assortment of different ways. They are an important source of unique polysaccharides (agar, carrageenan, alginates, *etc.*) for the pharmaceutical and food industries and the use of seaweed extracts as gelling agents and thickeners goes back almost half a millennium. The practice of extracting agar from seaweed was first described in 1658 in China and agar is well known today as a common substrate in bacterial culture medium, being first used by the pioneering German microbiologist Robert Koch [1]. Drift seaweed washed up on shore has been used as an organic agricultural fertiliser in coastal regions of the world for centuries. The application of seaweed as a fertiliser improves soil structure, provides trace elements and growth activators [4] as well as resulting in earlier seed germination, the enhancement of crop performance and yield and a better resistance to both biotic and abiotic stresses [5].

Since ancient times, edible seaweed species have formed an important part of the culinary tradition in countries of the Far East, such as China, Korea and especially Japan [6]. Seaweed is a very healthy food source with some varieties containing higher levels of minerals and trace elements than terrestrial plants and animal products [7,8,9,10]. For examples, a 100 g portion of seaweed can exceed the RDA value for vitamin A, B2 and B12 and two thirds of the vitamin C daily requirement [11]. The protein content of seaweed can vary greatly depending on many factors, such as the season when the plant is harvested and surrounding environmental conditions. Species of red seaweed can contain as much as 21–47 g/protein/100 g dry weight, while brown seaweeds have a comparatively low protein content of 7–16 g/100 g dry weight [12]. Seaweeds are also the best natural source of iodine and their addition to the diet could help people who are lacking in iodine to meet their daily iodine requirements [13]. In traditional Japanese cooking, edible seaweeds are extensively used as a sea vegetable and can also be used as condiments, seasonings and wrappings for sushi [14]. In such ways, they can account for as much as 25% of the daily food intake of some Japanese people [15]. Indeed, every year, over 1.6 kg of dry seaweed is consumed in Japan, on average per person [10]. Some of the more common seaweeds used in food preparation include the brown seaweed species, *Laminaria* (kombu), *Undaria* (wakame) and *Hijiki* (hiziki), and varieties of the red seaweed, *Porphyra* (nori). Edible seaweeds contribute few calories to the diet, owing to their low fat content and because seaweed derived carbohydrates and proteins cannot be fully digested in the gut by human intestinal enzymes [16]. As such, seaweeds are a good source of dietary fibre, which can positively affect satiety in between meals and glucose uptake from food [17]. Furthermore, soluble polysaccharide found in seaweeds may have a prebiotic effect, by stimulating the growth and/or activity of beneficial members of the microbiota such as the *Bifidobacterium* and *Lactobacillus* [18]. While seaweeds have undoubtedly been used extensively for thousands of years in Asia, South America and Oceania as a food source, the culinary use of seaweed has traditionally been very limited in both Europe and North America [1]. Despite this, the use of seaweed as sea vegetables has become more common in Western countries in recent decades [19] as a result of increasing globalization and improved accessibility of Asian cuisine to the rest the world. Furthermore, consumers in developed Western countries are increasingly turning to products from natural sources, including seaweeds [8,20].

Seaweeds are consistently exposed to both biotic and abiotic pressures in their natural marine environments. These pressures exert an influence on the plant’s physiology that leads to the production of metabolites in order for the plant to survive and thrive. Some of these metabolites may act as bioactive components, and thus have potential for use in the development of new functional ingredients and medical treatments. Indeed, secondary metabolites known to be produced by seaweeds have demonstrated therapeutic properties including anti-cancer, anti-oxidant, anti-inflammatory, and anti-diabetic activities [16]. Historically, Asian civilizations have used seaweeds for various medicinal purposes by boiling the seaweed in water and using the decoction as a drug. Japanese and Chinese practitioners have been recorded using seaweeds in herbal medicines as far back as 300 BC. The range of ailments reported to have been treated with seaweed or seaweed derived products is much varied. They include treatments for cancer, digestive problems, dropsy, eczema, glandular problems, goitre, gout, hyper-throidisms, parasitic infection, swollen and painful scrotum and urination and renal disorders [21,22,23]. In this regard, these metabolites may potentially lead to useful leads in the development of new functional ingredients and medical treatments [16].

**Figure 2:**
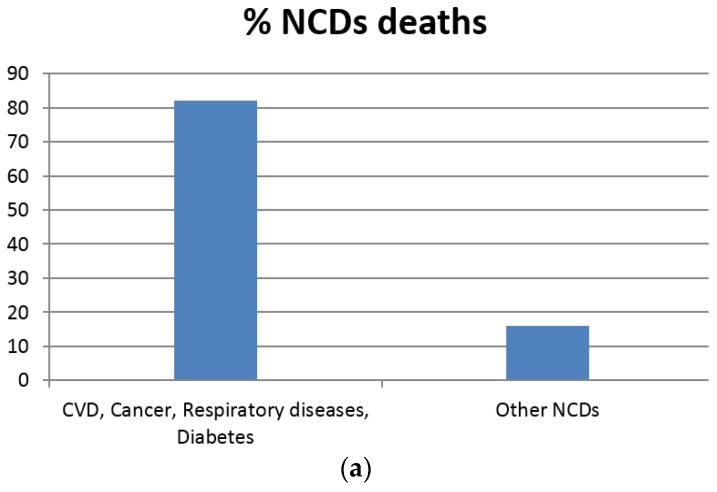

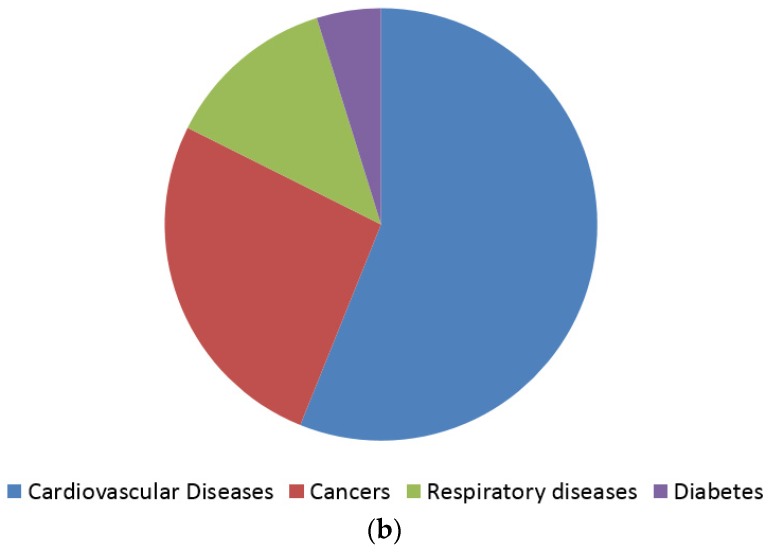
According to the World Health Organization (WHO) (**a**) cardiovascular diseases, cancers, respiratory diseases and diabetes account for 82% of all deaths attributed to non-communicable diseases; (**b**) Each year, cardiovascular diseases account for 17.5 million deaths, cancers (8.2 million), respiratory diseases (4 million) and diabetes (1.5 million) [24].

The aim of this review is to examine the literature with regard to the use of seaweed derived bioactive metabolites in relation to the treatment/prevention of a particular set of diseases referred to as chronic non-communicable diseases (NCDs). NCDs are an extensive group of conditions that, unlike bacterial and viral infections, are not transmissible from person to person. NCDs are a leading cause of death and disability, and affect millions of people globally each year. These long-lasting conditions have a protracted duration time and a generally slow rate of progression. The four main types of chronic NCDs are cardiovascular diseases (CVDs), cancer, diabetes mellitus and chronic respiratory ailments [24]. Chronic diseases typically begin to manifest in middle age, following long term exposure to a plethora of unhealthy activities, such as excessive alcohol consumption, primary and secondary smoke inhalation, low levels of physical activity, and the consumption of a diet with excess fat and red meat and low in fibre. The incidence of chronic diseases rises sharply as people start to age, with the majority of people over the age of 65 having a chronic ailment of one sort or another. Today, NCDs are the leading cause of death and disability in the world (Figure 2) and are responsible for double the sum total of deaths caused by all infectious deaths (including HIV/AIDS, TB and malaria), maternal and perinatal conditions and nutritional deficiencies [25]. As a consequence, interventions to prevent and control NCDs are essential and since seaweed is an abundant and natural resource with proven therapeutic effects, its contribution to the alleviation of chronic diseases are evaluated henceforth.

## 2. Anticancer Activity of Seaweed Components

Cancer is a generic name given to a large group of clonal malignant diseases. The defining characteristic of cancer is the rapid creation of abnormal cells that proceed to grow beyond their normal boundaries, leading to the invasion of adjoining areas of the body culminating in the spread of the cancer in a process termed metastases. Despite a better understanding of cancer biology in the last few decades, the treatment of most cancers has not progressed, with the reduction in cancer deaths mainly being attributed to early detection and preventive measures, rather than new cancer treatments coming on stream [26,27]. The challenge of developing effective treatments for cancer has encouraged the development of new drugs from natural sources, with seaweeds and the marine environment as a whole expected to be a major frontier in both pharmaceutical and medical cancer research [28].

### 2.1. Seaweed-Derived Polysaccharides

**Figure 3:**
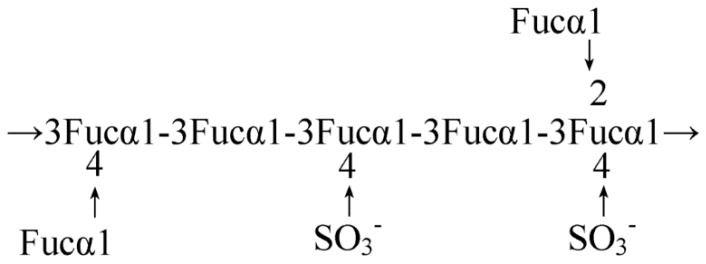
Model for the average structure of fucoidan from *Fucus vesiculosus*. The core region of the fucan is composed primarily of a polymer of α (1-3) linked fucose with sulphate groups substituted at the 4 position on some of the fucose residues [35].

Seaweeds, especially brown seaweeds, are rich in biologically active polysaccharides that exhibit a broad spectrum of biological activities. Examples of these polysaccharides include fucoidans, laminarins and alginic acids [29]. Fucoidans (fucans) are highly sulphated cell-wall polysaccharides found in species of brown seaweeds. Each different brown seaweed species produces its own array of fucans that have unique structural properties, which can be further altered by any number of biotic and abiotic factors to which the seaweed is exposed, as well as the extraction and purification method used to collect the fucan [30]. The biological activity of fucoidans is related to their molecular structure, which include fucose linkage, the sugar type, sulphate content, with molecular weight being the most important determinant. Fucoidan from *Fucus vesiculosus* (Phaeophyceae) is mainly composed of α-(1-3) linked sulphated l-fucose (Figure 3). In *Ascophyllum nodosum* (Phaeophyceae), α-(1-3) linked fucose with low proportion of α-(1-4) linked fucose or a repeating α-(1-3) and α-(1-4), has been reported. Linkages of α-(1-3) found in other polysaccharides has a stronger anticoagulation ability than the α-(1-4) configuration. The sulphate content of fucoidan also influences the anti-cancer and anticoagulant activities. Over sulphated fucoidan has a better α-amylase inhibitory activity than native fucoidan. Furthermore, the location of a sulphate group on fucose could also affect the biological function of fucoidan. A molecular weight that is too high may result in low solubility and processability, resulting in poor penetration of the cell. Low molecular weight (LMW) fucoidan degraded by gamma-irradiation was shown to increase cell cytotoxicity in comparison to native fucoidan in cancer cell lines such as AGS, MCF-7 and HepG-2. Gamma irradiated fucoidan also showed a higher level of cell transformation inhibition, resulting in higher anti-carcinogenic activity [31,32]

**Figure 4:**
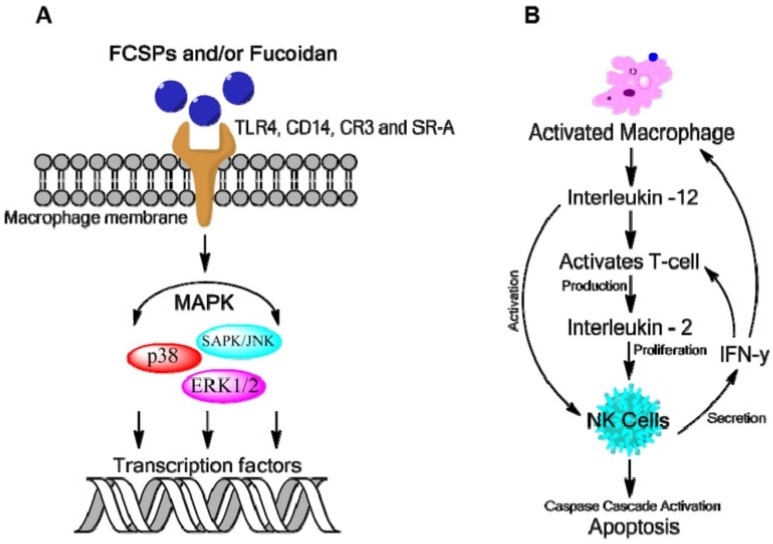
Proposed mechanism for fucoidan bioactivity (**A**) Macrophage activation by fucoidans as mediated through specific membrane receptor activation namely TLR-4, CD14, CR-3 and SR which induces intracellular signaling via mitogen-activation protein kinases (MAPKs); (**B**) Activation of macrophages leads to the production of cytokines such as IL-12, IL-2 and IFN-γ which enhance NK cell activation that may stimulate T-cell activation [41].

Evidence suggests that fucoidan can act as an anti-cancer agent through modulation of the human immune system. Fucoidan has been found to induce the maturation of dendritic cells and, in association with other cytokines, to shape the immune responses that are mediated by T-cells. For information of the proposed mechanism of fucoidan bioactivity see Figure 4. Dendritic cells are antigen-presenting cells that play a vital role in effectively stimulating the immune response as they are responsible for the initiation and polarization of adaptive immunity. Data suggest that fucoidan can modulate dendritic cell differentiation and drive it towards a Th1-polarizing phenotype, which could possibly be used in dendritic cell based vaccines for cancer immunotherapy [33]. Polysaccharides isolated from plants and algae have been reported to enhance macrophage activation through specific membrane pattern recognition receptors. These receptors recognize foreign ligands such as those found on carbohydrates during the innate immune response. The major receptors reported for polysaccharide recognition in macrophages are Toll-like receptor 4 (TLR4), CD14, complement receptor type 3 (CR3) and scavenger receptor (SR). Acetyl fucoidan isolated from commercially cultured *Cladosiphon okamuranus* (Phaeophyceae) induced macrophage activation in the murine macrophage cell line, RAW 264.7 through membrane receptors TLR4, CD14 and SRA (anti-scavenger receptor class A) and MAPK signaling pathways [34].

Fucoidan has also been shown to have cyto-protective properties. Chemotherapeutic anticancer drugs are effective against cancer cells but, because of a lack of selectivity, they also attack normal immune cells. It has been demonstrated that fucoidan can protect dendritic cells from the effect of 5-Fluorouracil (a representative cancer drug) [36]. Studies performed *in vitro* with crude fucoidan extracted from *Sargassum* sp. and *F. vesiculosus* demonstrated a reduction of the viable number of Lewis lung carcinoma cells and melanoma B16 cells in a dose dependent manner. Exposure to the fucoidan also caused morphological changes in the melanoma cells, which were indicative of apoptosis being induced. When male mice were challenged with daily i.p. injections of crude fucoidan from either seaweed over a 4 day period, the cytotoxic activity of their natural killer cells was enhanced, [37]. Fucoidan extracted from the sporophyll of *Undaria pinnatifida* (Phaeophyceae) was reported to show anti-tumour activity against PC-3, HeLa, A549 and HepG2 cell lines, which was comparable to that of commercially obtained fucoidan [38]. Fucoidan from *Saccharina cichorioides* (Phaeophyceae), *Fucus evanescens* (Phaeophyceae), and *U. pinnatifida* was investigated for effects on proliferation, neoplastic formation, and colony formation of mouse epidemial cells (JB6 C141), human colon cancer cells (DLD-1), breast cancer cells (T-47D) and melanoma (RPMI-7951). These particular fucoidans specifically and significantly suppressed the proliferation of human cancer cells and exhibited less cytotoxicity towards normal mouse epidermal cells [39]. Another investigated the possibility of using acetylated fucoidan (AcFu) nanoparticles loaded with the chemotherapy drug doxorubicin for the treatment of cancer using the cell lines HCT-116 and HCT-8. The nanoparticles demonstrated first-order drug release for 5 days following treatment. Treated macrophages were found to overexpress various anti-tumour cytokines, such as TNF-α and GM-CSF. The AcFu particles were also resistant to the multidrug resistant characteristics of cancer cells [40].

The laminarins are a group of water soluble polysaccharides produced by brown seaweeds. They consist of 1,3- and 1,6-linked β-d-glucose residues and normally have a molecular weight of 4–5 kDa. Laminarin isolated from *Eisenia bicyclis* (Phaeophyceae) was shown to inhibit human melanoma SK-MEL-28 and colon cancer DLD-1 cells. It was also demonstrated that decreasing the molecular weight of the laminarin (DP: 9–23) and increasing the ratio of 1–6 linked glucose residues increased the anticancer activity [29]. Rats fed a diet of 2% (*w*/*w*) laminarin suppressed indole, p-cresole and sulphide production significantly. Such compounds are produced from proteins by colonic bacteria and are putative risk markers for the development of colon cancer [42]. Other studies have also reported anti-cancer activity of laminarins and fucoidans [43]. One investigated the effectiveness of using polysaccharides from the edible *Saragassum latifolium* (Phaeophyceae) in chemoprevention. Fractions of water soluble polysaccharides from *S. latifolium* were tested for their chemopreventive efficacy revealing a range of chemopreventive properties, including anti-initiating, anti-promoting, and inhibition of NO, TNF-α and COX-2 [44]. A hot water soluble polysaccharide from *Capsosiphon fulvescens* (Chlorophyta) showed significant inhibition of human cancer cells in a dose dependent manner. Treated cells exhibited a marked increase in caspase-3 activation, and decreases in both the expression of Bcl-2 and the phosphorylation of insulin-like growth factor-I (IFG-1) receptor. Treatment with the polysaccharide extract also decreased the recruitment of p85 to IGF-1 receptor and insulin receptor substrate-1 (IRS-1) [21]. Sulphated polysaccharides from the thallus of *Sargassum plagiophyllum* (Phaeophyceae) were shown to have anti-cancer activity against HepG2 and A549 cell lines [45]. Porphyrans from Porphyra species induced cell death in human AGS gastric cancer cells in a dose dependent manner by decreasing cell proliferation and inducing apoptosis [46]. Carrageenan extracted from *Solieria chordalis* (Rhodophyta) showed no cytotoxicity towards human cancer cells lines but demonstrated immune-stimulating properties. Treatment resulted in enhancement of neutrophil phagocytosis, cytotoxicity by natural killer cells, antibody-dependent cell cytotoxicity and stimulation of lymphocyte proliferation, which points towards a use in cancer immunotherapeutic treatment [47]. A heterofucan isolated from *Spatoglossum schröederi* (Phaeophyceae), Fucan B, was found to inhibit the proliferation and migration of CHO-K1 when fibronectin was used as the substrate. Fucan B also promoted G_1_ cell cycle arrest [30]. A summary of recently reported biological activities found in algal polysaccharides are outlined in Table 1.

### 2.2. Fatty Acids

Fatty acids are commonly found in foods such as vegetable oils, meat, milk, and soy products. They play an important role in maintaining normal physiological functions. Docosahexaenoic acid (DHA) and arachidonic acid (AA) are both important parts of mammalian cell membranes and are crucial to brain and eye development in human infants. The intakes of omega-3 and omega-6 fatty acids have been linked to a reduction in cardiovascular mortality rates, suppression of arthritis-associated inflammation, and a decreased risk of cancer. Marine algae such as seaweed are a rich source of unsaturated fatty acids. An isolated diketosteroid, (*E*)-stigmasta-24(28)-en-3,6-dione (Compound 1) along with three previously known steroids from *Tydemania expeditionis* (Chlorophyta)*,* namely β-sitosterol (2), fucosterol (3) and saringosterol (4) collected from the China sea were evaluated for activity on prostate cancer cell lines DU145, PC3 and LNCaP. The diketosteroid termed (compound 1), showed moderate inhibitory activities while fucosterol proved to be most effective. Two unsaturated fatty acids isolated from a Fijian population of the *T. expeditionis,* were shown to have moderate inhibitory activity against a panel of tumour cell lines (including breast, colon, lung, prostrate and ovarian cells [59].

### 2.3. Carotenoids and Terpenes

Carotenoids are natural tetraterpenes which are produced by a wide variety of organisms ranging from single celled microbes to plants with more than 700 examples described so far [60]. The carotenoid β-carotene, which is found in large quantities in green and yellow fruit, and lycopene are both known for their anti-cancer activities [61]. Fucoxanthin is a carotenoid that is found in great abundance in Brown seaweeds [62]. Indeed, it is the most abundant of all carotenoids, accounting for more than 10% (approximately 10 million tonnes) of the estimated natural production of carotenoids each year [63]. Fucoxanthin is reported to be very effective in inducing cellular death in human leukaemia and colon cancer cells [64] and has been proven to suppress *in vivo* liver and skin carcinogenesis [61]. The ability to scavenge free radicals is thought to play an important role in the anti-mutagenic and anti-carcinogenic mechanisms of carotenoids, and as such, fucoxanthin displays potent scavenging abilities. To date, literature pertaining to the anti-cancer activity of carotenoids in seaweeds has focused mainly on that of fucoxanthin. However, the exact mechanism by which fucoxanthin exerts it anti-cancer activity has not yet been fully defined. Fucoxanthin can strongly and concentration-dependently inhibit the growth of human hepatoma cells after 24 h, and facilitate the growth of mouse embryonic cells at the same time. Fucoxanthin was able to significantly enhance gap junctional intercellular communication (GJIC) of the cancer cells without affecting noncancerous mouse cells. Treatment with fucoxanthin also resulted in an increase in both protein and mRNA expression. The upregulation of GJIC, coupled with increases in intracellular calcium levels may be responsible for cell cycle arrest and cellular death via apoptosis [63]. Fucoxanthin derived from seaweed *Undaria pinnatifida* (Phaeophyceae) was shown to markedly reduce the viability of different colon cancer cell lines *in vitro*. Treatment induced DNA fragmentation and reduced the level of the anti-apoptotic protein, Bcl-2. It was also noted that separate treatment of CaCo-2 cells with fucoxanthin and troglitazone recorded no decrease in cell viability, but when used in combination, cell viability was greatly reduced [62]. Fucoxanthin was shown to inhibit tumour cell growth in HepG2 cells by inducing G1 cell cycle arrest and/or inducing apoptosis [65]. In nature, the majority of carotenoids occur predominately or entirely in the trans-form. The presence of a cis double bond garners greater steric hindrance between close-by hydrogen atoms and/or methyl groups, resulting in a bond that is less thermodynamically stable than the trans-form. The all-trans form of fucoxanthin was the major geometrical form found in the sample investigated. However, a mixture of 13-*cis* and 13′-*cis* isomers produced the strongest anti-proliferative activity of all the geometrical isomers [64]. Fucoxanthin from *Saccharina japonica* (formerly *Laminaria japonica*) (Phaeophyceae) has been shown to suppress the invasion of highly metastatic B16-F10 melanoma cells. This form of fucoxanthin inhibited the expression and secretion of MMP-9, which plays a critical role in tumour invasion and migration. Furthermore, the expression of cell surface glycoproteins that play an important role in migration, invasion and cancer-endothelial cell adhesion was diminished. In lung cancer metastasis models, fucoxanthin caused a significant reduction of tumour nodules [66].

Another carotenoid of interest is siphonaxanthin, which is a keto-carotenoid found in siphonaceous green algae. In comparison with other carotenoids such as fucoxanthin, siphonaxanthin is a potent inhibitor of HL-60 cells. Treatment with siphonaxanthin resulted in a significant reduction in cell viability within 6 h. An increase in TUNEL-positive cells and chromatin condensation in the HL-60 cells indicated apoptotic activity. The induction of apoptosis also reduced the expression of Bcl-2 and increased the expression of caspase-3 [67]. Halogenated monoterpenes are produced by marine algae of the families Plocamiaceae and Rhizophyllidaceae and have a well-established anticancer potential. Four halogenated monoterpenes isolated from *Plocamium suhrii* (Rhodophyta) exhibited greater cytotoxicity when compared to cisplatin, a known anticancer drug, when assayed against an esophageal cancer cell line [68]. Polyhalogenated monoterpenes from *Plocamium corallorhiza* (Rhodophyta) also showed moderate cytotoxicity towards esophageal cancer cells [69] Peyssonoic acids A–B and a novel sesquiterpene hydroquinnones isolated from Peyssonnelia sp. exhibited modest antiproliferative activity against ovarian cancer cells [70].

### 2.4. Seaweed Derived Antioxidants

Reactive oxygen species (ROS) are highly reactive molecules that are constantly produced by cellular enzymatic reactions. They are required to maintain cell homeostasis and the body’s antioxidant defence systems are designed to prevent harmful effects caused by increased levels of ROS. Cells in a normal healthy condition produce ROS at low levels. Free radical-mediated modification of DNA, proteins, lipids and small cellular molecules have been associated with such diseases as cancer, atherosclerosis and rheumatoid arthritis [71]. Antioxidants are secondary metabolites that inhibit oxidation by transforming radicals into non-radicals by donating electrons and hydrogen, chelating transition metals and dissolving generated peroxidation compounds. The antioxidant compounds produced by plants include phenolic compounds such as flavonoids, cinnamic acid, benzoic acid, gallic acid, phlorotannins and quercetin.

Among marine organisms, seaweeds represent one of the richest sources of antioxidants [72]. In South East Asia, *Eucheuma cottoni* (Rhodophyta) is grown in abundance for human nutrition. A polyphenol rich extract from *E. cottoni* was shown to be anti-proliferative against oestrogen-dependent MCF-7 and oestrogen-independent MB-MDA-231 human breast-cancer cells *in vitro*, but non-toxic to non-cancerous cell lines. The extract was fed to female rats and following four weeks of dietary supplementation, mammary tumours were induced with carcinogenic agents. Tumour development and erythrocyte lipid peroxidation was inhibited in rats that had previously received the extract as well as induction of mammary tumour apoptosis, down-regulation of oestrogen biosynthesis and an improved antioxidant status [73]. Soluble fractions of *Palmaria palmata* (Rhodophyta), *Laminaria setchellii* (Phaeophyceae), *Macrocystis integrifolia* (Phaeophyceae) and *Nereocystis leutkeana* (Phaeophyceae) have been shown to inhibit the proliferation of HeLa cells. The anti-proliferative effect of the seaweed extracts was positively linked to their total phenolic content [15]. One of the key antioxidant defence mechanisms in the cell is the NF E2-related factor 2 (Nrf2)—antioxidant-response element (ARE) signalling pathway, which can be activated by a variety of small molecules. Fractionation of the edible seaweed *Ulva lactuca* (Chlorophyta) gave rise to multiple active fractions as measured by an ARE-luciferase reporter assay. A keto-type C18 fatty acid was shown to induce the expression of cytoprotective genes with its cellular activity requiring the presence of Nrf2 and PI3k function. Mice treated with a single dose of an *U. latuca* fraction that was enriched with the C18 fatty acid showed similar ARE-activating effects to those observed in *in vitro* studies. This observation could be due to the ability of the fraction to inhibit KEAP1-mediated Nrf2 ubiquitination and the subsequent accumulation and nuclear translocation of Nrf2. A significant increase in the transcript levels of *Nqo*1 was also found in other mouse tissues such as the brain, stomach and lung [74].

Fucoidan has also been shown to exhibit antioxidant activity. When different sulphated polysaccharides from the seaweed *Turbinaria conoides* (Phaeophyceae) were evaluated for antioxidant activity, fucoidan showed the highest antioxidant potential followed by alginic acid and laminarin, respectively [71]. The anti-cancer properties of *Laurencia obusta* (Rhodophyta) were correlated with its total phenolic and flavonoid contents [75]. Polyphenol rich extracts from *Ecklonia cava* (Phaeophyceae) have shown strong anti-cancer activities. One study demonstrated significant suppression (*p* > 0.05) of migration and invasion of A549 cells in a dose-dependent manner and down regulation of the matrix metalloproteinase (MMP)-2 activity, which is essential in the degradation of the extracellular matrix [76]. Another poly-phenolic rich fraction from *E. cava* exhibited strong selective cell proliferation inhibition on all cancer cell lines tested (CT-26, THP-1, B-16 and U-937), which was attributed to induced apoptosis in CT-26. The extract also demonstrated strong radical scavenging activity and reducing power and at 5µg/mL was found to be comparable to butylated hydroxytoluene at the same concentration [77]. Also, phloroglucinol derivatives from *E. cava* inhibited MCF-7 human cancer cells proliferation apoptosis triggered through NF-κB family and NF-κB dependent pathways [78]. Oxidative stress brought about by long term exposure to ultraviolet radiation from sunlight plays an important role in the development of skin cancer. Ultraviolet B radiation in particular (by having a longer wavelength 280–320 nm) is associated with a more harmful impact on the skin. Protective compounds against biotic factors such as UV radiation has been produced by *Undaria crenata* (Phaeophyceae), with ethanol extractions having demonstrated photoprotective activity against cell damage caused by exposure to UVB radiation in Human HaCaT keratinocytes. Analysis revealed a significant scavenging effect of the extract against superoxide anion and hydroxyl radical. UVB-induced apoptosis was reduced, resulting in recovery of cell viability. Treatment also decreased the level of UVB-induced oxidative stress to lipids, proteins, and DNA, as shown by a decrease in the level of 8-isoprostane, protein carbonylation and DNA tails [79].

### 2.5. Anti-Cancer Activity of Minor Seaweed Components

It is well documented that major seaweed components such as fucoidan and fucoxanthin have effective anti-cancer properties. However, the importance of screening crude seaweed extracts should not be overlooked, as minor components may also harbor potent biological activities. The sporophyll of *U. pinnatifida* is considered to have lower utility value compared to other parts of the plant and is usually discarded as waste. An ethanol extract of the sporophyll was prepared and shown to reduce the viability of colorectal cancer HCT116 cells [80]. A novel glycoprotein isolated from *S. japonica* (formerly *L. japonica)* (LJGP) was found to have anti-proliferative effects on numerous cancer cell lines in a dose-dependent manner. LJGP treatment of HT-29 cancer cells caused them to display several apoptotic features such as DNA fragmentation, sub-G1 arrest, caspase-3 activation, and Poly (ADP-ribose) polymerase (PARP) degradation. It was also determined that LJGP-induced apoptosis led to the formation of a death-inducing signalling complex (DISC) of Fas, FADD and procapase-8. LJGP induced the reduction of mitochondrial membrane potential with the activation of the Bcl-2 family of proteins and caspase-9 [81]. The enzyme telomerase adds tandem arrays of TTAGGG repeats to the ends of telomeres. Telomerase activity is not usually detectable in normal cells but high activity is found in most cancer cells. As a result, telomerase represents a promising target for cancer therapy and much work has been performed on screening for telomerase inhibitors. Eitsuka *et al.*, (2004) [82] confirmed the inhibitory effect of sulfoquinvosyldiacylglycerol (SQDG), a glyceroglycolipid, from *Porphyra yezoensis* on human telomerase in a cell-free system, which acted in a dose-dependent manner. It was also confirmed that EPA, which is a component of SQDG, is a potent telomerase inhibitor.

Three pigments isolated from an extract of *Porphyra tenera* (Rhodophyta) (β-carotene, chlorophyll a and lutein) showed significant activity against mutagen-induced *umu* C gene expression. Combined treatment with the pigments showed an additive effect compared with single treatment with each pigment [83]. The same authors later studied the *in vivo* anti-carcinogenic activity of the seaweed *Ulva prolifera* (formerly *Enteromorpha prolifera*) (Chlorophyta) using an initiator (7, 12-dimethylbenz[*a*]anthracene) and promoter (12-*O*-tetradecanoylphorbol-13-acetate) model. The application of *U. prolifera* extract prior to initiator or promoter treatment caused a significant suppression of mouse skin tumourigenesis. The combined use of the extract before both treatments (with initiator and promoter) resulted in much stronger suppression against the same skin tumourigenesis. It was proposed that a chlorophyll-related compound, pheophytin-a was an antigenotoxic substance [84]. The anti-tumour effect of pepsin-digested *Caulerpa microphysa* extracts was demonstrated by their addition to HL-60 and WEHI-3 cell lines. The growth of both cell lines were significantly affected (*p* < 0.05) when incubated with the digested extract at concentrations of 25 µg/mL and above. A significant increase in DNA damage was also recorded at concentrations of 100 µg/mL and above in comparison with the control cells [85].

## 3. Potential of Seaweed Components to Alleviate Cardiovascular Disease

Cardiovascular diseases (CVD), including heart disease and stroke are a diverse group of disorders that affect the mammalian circulatory system. Collectively, CVDs are the number one cause of human death worldwide. In 2008, 30% of all deaths were as a result of a CVD. Although many risk factors for CVDs are recognised, the most important are hypertension, hyperlipidemia, hyperglycemia and abdominal obesity [86]. The traditional Japanese diet, which is characterised by high consumption of fish, seaweed and other plant material and sodium, with an accompanying decrease in refined carbohydrates and animal fat has been associated with a reduced risk of mortality associated with CVD. Today, Japan enjoys one of the lowest rates of coronary heart disease of any country in the world [87].

### 3.1. Hypertension and Hyperlidemia

Hypertension or high blood pressure is a major modifiable risk factor of cardiovascular disease. Known as the “silent killer”, hypertension can be asymptomatic for years before the condition is diagnosed clinically [88]. Cases of hypertension are divided into those of essential, primary or idiopathic hypertension with essential hypertension accounting for 95% of all cases. Risk factors that contribute to the development of hypertension include differing concentrations of sodium and potassium in the body, obesity, resistance to insulin, high alcohol intake, low calcium intake, stress and ageing. Many of these factors, such as being obese and having a high alcohol intake are additive over time. Approximately 25% of the global adult population suffers from hypertension, with this percentage expected to reach 60% of the population by 2025 [89,90]. The prevalence of high blood pressure increases as people age. Indeed, in developed countries, 65% of those aged between 65 and 74 are affected by hypertension. Diet and lifestyle modifications are most often used to lower blood pressure levels [91].

Potassium alginate is a major polysaccharide present in brown seaweeds. Alginates are known to have the ability to bind sodium, potassium and calcium ions and decrease the absorption of sodium in the intestine resulting in reduced blood pressure. In this regard, dried seaweed flakes containing potassium alginate could be used as a replacement for table salt for people with high blood pressure [90,92]. An epidemiological study performed in 25 countries spanning 15 years concluded that changes in dietary patterns such as cutting back on salt, increasing the consumption of fish oil, soybean protein and dietary fibre (including from seaweed) could reduce the risk of suffering a stroke [93]. Research into the effect of sulphated polysaccharides from *S. japonica* (formerly *L. japonica)* on rats with induced vascular endothelial damage after a psychological stress (PS) showed that adrenalin metabolites in plasma were significantly lowered in rats after administration of the seaweed extract. It was shown that the polysaccharide extract had a vascular endothelial cell-protective effect in stressed rats [94]. A low-molecular weight alginate extracted from *L. japonica* was shown to decrease systolic blood pressure in hypertensive rats. Rats that had high blood pressure induced displayed increased systolic blood pressure, sodium excretion, serum sodium and potassium levels, circulating plasma volume (CPV) and plasma atrial aldosterone (ALD) compared to a control group of non-induced rats. Treatment with the alginate extract normalised the induced changes. Furthermore, forms of potassium that do not contain chloride might offer better cellular entry in exchange for sodium and augment anti-hypertension activity [90]. Wakame powder from *U. pinnatifida* (5% *w*/*w* in a diet) significantly delayed signs of stroke and the survival rate of salt loaded, spontaneously hypertensive stroke-prone (SHRSP) rats [95]. In an early trial attempting to decrease sodium intake and increase potassium intake, a group of middle-aged patients suffering from mild hypertension were given a seaweed preparation (potassium loaded, ion-exchanging, sodium—adsorbing and potassium releasing). After four weeks of dietary intervention, there was a significant decrease in the mean blood pressure of those taking 12 and 24 g/day of the preparation [96]. While hypertension is mainly associated with adults, many studies have tracked blood pressure from childhood to adulthood with some showing that the process of atherosclerosis begins in childhood. Thus, monitoring blood pressure from an early age and appropriate intervention is important in preventing the development of CVD in later life. A study undertaken amongst Japanese preschool children examined the effect that seaweed intake had on blood pressure levels. Seaweed intake was significantly negatively related to systolic blood pressure in girls and negatively related to diastolic blood pressure in boys suggesting that seaweed as part of the diet might have beneficial effects on blood pressure among children [97].

Hyperlipidaemia is a major cause of CVDs by bringing about sustained endothelial dysfunction and vascular inflammation [98]. A diet of restructured pork enriched with *Himanthalia elongata* fed to rats reduced plasma cholesterol levels in test subjects that were supplemented with dietary cholesterol [99]

### 3.2. The Renin-Angiotensin System

The renin-angiotensin system (RAS) is a major regulator of blood pressure and fluid homeostasis in the body. Disruption of the RAS system can lead to increased blood pressure and the development of cardiovascular disease, chronic kidney disease and diabetes [100]. The two key enzymes in the RAS system are renin and angiotensin converting enzyme 1 (ACE-1). The inhibition of ACE-1 is a favoured strategy in treating hypertension [101] and several synthetic ACE inhibitors (captopril, lisinopril, enalapril and fosinopril) are thus used for this reason in the treatment of hypertension [102] Despite their effectiveness, synthetic ACE inhibitors are responsible for a number of unpleasant side effects such as development of a cough, loss of taste, renal impairment, and angioneurotic oedema and as a consequence there has been a trend recently to explore and develop more natural inhibitors of ACE activity [88]. Several studies have investigated the ACE inhibitory potential of compounds isolated from *U. pinnatifida*. Administration of the *U. pinnatifida* peptide led to a significant decrease in blood pressure in spontaneously hypertensive rats [103,104]. A cold water protein extract derived from *Porphyra columbina* (Rhodophyta) has been shown to have antihypertensive properties (>35% of ACE inhibition) [105]. *C. microphysa* pepsin digested extracts were determined to have greater ACE inhibitory activity than Flavourzyme or Alcalase [85]. Using enzyme hydrolysis, highly functional antihypertensive peptides have been produced from *Porphyra yezoensis* (Rhodophyta) Peptides produced under optimal conditions (1.5% substrate, 5% alcalase, pH 9.0, temperature of 50 °C and hydrolysis time of 60 minutes) had high antihypertensive activity (55% of ACE inhibition and a low IC_50_ value of 1.6 g/L [106]. A protein hydrolysate from *P. palmata* with *in vitro* renin inhibitory properties baked in bread was found not to have affected the texture or sensory properties of the bread to a large degree. The bread containing the hydrolysate also retained the renin inhibitory activity following preparation and could represent a new method for the delivery of renin inhibitory substances [101].

### 3.3. Heart Disease

Heart attack (myocardial infarction) is the leading cause of death for both sexes across the globe. It occurs when there is an insufficient blood supply to the myocardium leading to death of the myocardial muscle (ischemia). Prolonged ischemia leads to necrosis which is also termed myocardial infarction. Fucoidan from *C. okamuranus* was evaluated in rats where myocardial infarction was induced by isoproterenol, a synthetic catecholamine that has been known to cause severe stress in the myocardium. Fucoidan reduced the induced myocardial damage and improved the antioxidant defence system, reducing oxidative stress [107]. Similarly the pre-treatment of isoproterenol induced myocardial injured rats with fucoidan from *T. conoides* saw a significant normalization of the endogenous and exogenous antioxidant defense system [108]. Heparin is a widely used anticoagulant that has unfortunate side effects such as bleeding and low platelet count (thrombocytopenia) as well as being potentially contaminated with prions and viruses from their animal sources. Fucoidans from algal sources are known to have anticoagulant activities and have been proposed as an alternative therapeutic treatment. Low and high molecular weight fucoidans were tested for their anti-aggregant, anti-coagulation and anti-thrombotic activities. When tested in the platelets of humans and rats, the high molecular-weight fucoidan showed pro-aggregation activity, whereas the low molecular-weight fucoidan demonstrated an inhibitory effect on thrombin induced aggregation, with an IC_50_ of 8 µg/mL, five-fold less than that of commercially available fucoidan or heparin. The inhibitory effects of low molecular-weight fucoidan and heparin on thrombin activity were greatly enhanced by either antithrombin or heparin cofactor II (HCII). Results indicated that low molecular weight fucoidan inhibits thrombin via activation of antithrombin and HCII, whereas commercial fucoidan mainly interacts directly with thrombin [109]. Other low molecular weight fractions of fucoidan from *S. japonica* (formerly *L. japonica)* were reported to also have strong anticoagulant activities [110].

### 3.4. Marine Derived Oils and Fatty Acids

Omega-3 oils are produced naturally by algae and phytoplankton which are then consumed by fish resulting in an accumulation of eicosapentaenoic acid (EPA) and docosahexaenoic acid (DHA) in their flesh [111]. Human populations with a high consumption of fish have an inverse relationship with coronary heart disease and breast cancer. This inverse relationship first came to light from epidemiological studies of Inuit and Japanese populations, both of whom have lower incidences of CVD and cancer. In their homelands, the traditional diet of both groups contained appreciable amounts of fish. As they migrate over time to other areas, they adopt local dietary patterns and the incidences of CVD and cancer among them increase to the level of the local native people [111]. Fish and marine derived oils such as those from seaweed are rich in the omega (ω)-3 oils, eicosapentaenoic acid (EPA; 20:5 ω-3) and docosahexaenoic acid (DHA; 22:6 ω-3). Nutritional compositional studies of *Laminaria* sp., *U. pinnatifida, Sargassum fusiforme* (formerly *Hizikia fusiformis*) (Phaeophyceae) and *Porphyra* varieties found that they contained high levels of these oils [112]. Essential fatty acids play an important role in many biological processes. Following absorption from the gut, fatty acids are incorporated into triglycerides, phospholipids and cholesterol esters. Phospholipids are needed for the formation of cell membranes in every cell in the body. Omega-3 oils are fatty acids that have a signature double bond at the third position from the methyl (omega) end of the molecule. Such fatty acids cannot be synthesised by humans as the required enzymes to introduce a double bond at the correct position are missing. Such oils must be ingested as part of the diet [113]. The fluidity of the cell membrane is of great importance for receptor function and signaling pathways. The level of fluidity is determined in part by the amounts of phospholipids and fatty acids in the membrane that have double bonds. Multiple double bonds increase the fluidity of cell membrane and may partially account for the health benefits of omega-3 oils in preventing cardiac arrhythmias, as well as maintaining neurological function. DHA comprises only 4% of the fatty acid contents in the bloodstream but is almost 30% of the fatty acids in the phospholipids in the brain and retina, implying an important role in neurological and visual function [113]. In the mammalian heart, both DHA and EPA are incorporated into the cell membrane of cardiomyocytes, the levels of which can be significantly increased by taking food supplements containing omega-3 fatty acids. EPA and DHA, released from myocardial membranes, exert anti-arrhythmic effects by prolonging the refractory periods of cardiac action potential. In a study of heart tissue from cadavers, levels of omega-3 and omega-6 fatty acids were found not to be associated with cardiac mortality. However, their presence in low levels (especially DHA and AA) were associated with high mortality in those with a history of coronary heart disease [114].

Ischemia-reperfusion injuries occur when tissues in the body are deprived of oxygen for a short period of time and the resumption of blood flow causes intense inflammation [115]. The intake of hydrogen gas has been shown to be an effective treatment for Ischemia-reperfusion injuries. Bacteria in the gut can produce hydrogen gas and it has been demonstrated that oral administration of mannitol to humans and animals can increase its production. Seaweed is a good source of mannitol and consumption can have a protective effect [116]. Seaweeds are also a good natural source of conjugated fatty acids (CFAs), isomers of PUFAs with a double bond in their structure. Dietary CFAs such as conjugated linoleic acids (CLAs) have been reported to prevent the onset of essential hypertension in non-obese hypertensive rats by regulating the production of physiologically active adipocytokines such as adiponectin, leptin and angiotensinogen [117].

## 4. Potential of Seaweed Components to Alleviate Diabetes Mellitus and Obesity

Diabetes mellitus is a chronic disease where the pancreas does not produce enough insulin or when the body cannot use the insulin it produces effectively. Insulin is a hormone required for cells to take up glucose from the blood. People with diabetes exhibit an altered glucose metabolism [118] The inability to utilize glucose properly results in progressive complications in various bodily functions, and affects mineral levels in the body [119]. The vast majority of cases of diabetes mellitus present as either Type-1 diabetes or Type-2 diabetes, with Type-2 diabetes accounting for 90% of reported cases [120]. Since abnormally high or low blood glucose levels can lead to chronic cardiovascular problems, chronic renal failure, nerve damage, fainting and diabetic coma, people with diabetes must monitor and keep their condition under control at all times. Products obtained from nature have a long tradition of use in the treatment of diabetes. For example, plants that have high polyphenol content have the ability to inhibit the activity of carbohydrate hydrolysing enzymes such as α-amylase and α-glucosidase. This has the effect of lowering postprandial levels of glucose [121]. Seaweeds contain many components that are believed to be beneficial in the treatment of diabetes [122].

### 4.1. Seaweed-Derived Antioxidants in Treating Diabetes Mellitus

In humans, a balance exists between the production of ROS and their inactivation as previously discussed. Oxidative stress comes about under certain conditions, such as *diabetes mellitus*, when the balance between production and inactivation is disrupted and ROS overwhelms the cell’s antioxidant capabilities. Oxidative stress is reported to be the basal cause for the development of insulin resistance, β-cell dysfunction, impaired glucose intolerance and full blown type-2 diabetes [123,124]. Several synthetic antioxidants, such as butylated hydroxyanisol (BHA), butylated hydroxytoluene (BHT) and tert-butylhydroquinone (TBHQ) are available commercially but their use is now being restricted owning to adverse side effects, including the promotion of cancerous cells [125]. Because of this, interest in using naturally obtained antioxidants for diabetic treatment has increased [126]. Seaweeds are generally considered to be a rich source of antioxidant compounds as previously discussed. Pigments such as fucoxanthin and astaxanthin, and polyphenolic compounds such as phenolic acid, flavonoid, and tannins have all exhibited antioxidant abilities [118]. Polyphenolic compounds can act as scavengers of ROS. However fundamental differences exist between the polyphenols produced by land plants and those produced by their marine counterpoints. This makes marine derived polyphenols a promising new target source for phenolic compounds that could be used as lead drugs in the pharmaceutical industry [123]. Edible seaweeds are a good source of polyphenols and by being able to modulate glucose-induced oxidative stress. Polyphenols are suggested to have anti-diabetic activity.

When extracting any potentially useful compound(s) from a source, it is important to remember that the profile of the extract is dependent on the profile of the solvent or solvents used. For example, ethanol and methanol can break down the plant cell walls more efficiently and are believed to be more effective at extracting antioxidant compounds from seaweeds than water [127]. The *T. conoides* showed higher total phenolic content (TPC) when developed as an extract in methanol, when compared to diethyl ether extract [128]. The brown seaweed *Tubinaria ornata* (Phaeophyceae) has demonstrated superoxide scavenging activity which may be effective in reducing the level of O_2_ that is elevated during oxidative stress in the body. The presence of phenolic compounds suggests that the antioxidant activity might be due to them [129]. Methanol extracts from *E. cava* containing high levels of polyphenol and strong ROS scavenging ability significantly reduced blood glucose levels and increased insulin concentration when fed to type-1 diabetic rats. Blood alanine transaminase (ALT) levels were dramatically reduced to near normal levels. Increased levels of ALT in serum are often associated with health problems such as diabetes and liver damage. The anti-diabetic effect appears to be at least partly mediated by the activation of both the AMP-activated protein kinase/ACC and the Pl-3 kinase/Akt signal pathways [130]. The addition of the edible seaweeds to foodstuffs may provide a useful method of enhancing their anti-diabetic qualities. Added *H. elongata, U. pinnatifida* and *Porhyra umbilicalis* (Rhodophyta) served as a source of soluble polyphenolic compounds in low salt meat emulsion model systems and increased the antioxidant capacity of the meat. The increased antioxidant load of the samples leads to greater stability during processing and storage of the meat [131]. Numerous other seaweeds have also been found to contain high amounts of phenolic compounds and exhibited strong antioxidant activity, these include *Sargassum swartzii* (formerly *Sargassum wightii*) [125]; *Fucus serratus* (Phaeophyceae) and *F. vesiculosus* [132].

### 4.2. Controlling Glucose Levels in the Blood

High levels of glucose in the blood following carbohydrate ingestion have an important role in the development of type-2 diabetes as well as in complications that rise from the disease. The control of postprandial hyperglycemia is of great importance in the treatment of diabetes and the prevention of cardiovascular complications. One avenue of treatment is to prevent the absorption of glucose by inhibiting carbohydrate-hydrolyzing enzymes such as α-amylase and α-glucosidase. Synthetic inhibitors of α-amylase and α-glucosidase such as miglitol, voglibose and acarbose are designed to sharply reduce the blood sugar level that spikes after meals. The use of these however has several undesirable side effects such as flatulence, abdominal cramps, vomiting and diarrhoea. Seaweeds are known to have the ability to inhibit starch digestive enzymes and are an underexplored source of enzymatic inhibitors for use in the treatment of diabetes [133,134]. A study investigating the α-amylase and α-glucosidase inhibitory effects of fifteen Irish seaweeds found that cold water and ethanol extracts of *A. nodosum* had a strong α-amylase inhibitory effect while extracts of *F. vesiculosus* exhibited potent inhibition of α-glucosidase. The recorded effects of the extracts were associated with phenolic content and antioxidant activity [135]. The phenol rich extracts of *A. nodosum* collected from UK waters have also been shown to inhibit α-amylase activity to some extent. In a study conducted with samples of *A. nodosum, P. palmata* and *Alaria esculenta* (Phaeophyceae), the *A. nodosum* extracts were found to be the most active of the three seaweeds. The same extracts were also able to inhibit the activity of α-glucosidase at low levels. Following fractionation of the Ascophyllum extracts, it was found that the inhibitory activity was concentrated in the phlorotannin rich fraction. It has been suggested that seaweeds accumulate phlorotannins to deter being eaten by predatory species such as molluscs and they have been shown to potently inhibit the digestive glycosidases of marine snails [136]. Two bromophenols (2,4,6-tribromophenol and 2,4-dibromophenol) isolated and purified from the red seaweed *Grateloupia elliptica* (Rhodophyta) were found to have high α-glucosidase inhibitory activity. In addition, both compounds mildly inhibited rat-intestinal sucrase and rat-intestinal maltase. Both sucrase and maltase are similar in activity to α-glucosidase in so much as they break down sucrose and maltose to glucose. The authors of this study concluded that the bromophenols of *G. elliptica* have potential as natural nutraceuticals to prevent diabetes mellitus [17]. Acetone crude extracts from *S. schroederi* and *Caulerpa racemose* (Chlorophyta) both inhibited α-amylase activity [137]. Dieckol isolated from *E. cava* showed pronounced α-amylase and α-glucosidase inhibition displaying higher activity than that of acarbose. Postprandial blood glucose levels in streptozotocin induced diabetic mice were also seen to be significantly suppressed [134]. Diphlorethohydroxyycarmalol (DPHC) isolated from *Ishige okamurae* (Phaeophyceae) showed strong inhibition of α-amylase and α-glucosidase without having any toxic effects on human umbilical vein endothelial cells (HUVECs) at various concentrations. In induced diabetic mice, extracts of *Petalonia binghamiae (*Phaeophyceae) (PBE) have been demonstrated to have anti-diabetic properties. Treatment with extract resulted in reduced blood glucose levels in diabetic mice and there was an improved tolerance to glucose [138]. Ethanol extracts from *Ulva rigida* (Chlorophyta) have been shown to have strong anti-hyperglycemic and antigenotoxic effects in diabetic mice [139].

### 4.3. Other Anti-Diabetic Activities

Numerous studies indicate that a diet enriched in whole, unprocessed plant foods that are abundant in phytochemicals may be of benefit for metabolic disorders such as diabetes. Obese mice supplemented with an *U. pinnatifida* ethanol extract showed significantly reduced amount of visceral fat, adipocyte size, fasting blood glucose concentration and plasma insulin after nine weeks compared to the high fat fed control group. Results indicated that insulin resistance and hepatic fat build-up can be prevented by modulating the hepatic glucose and lipid homeostasis in the high fat induced obese mice [140]. Mice fed extracts of *I. okamurae* for six weeks were found to have an improved blood glucose level and a lower level of blood glycosylated haemoglobin when compared to non-diabetic control mice. Data suggested that the *I. okamurae* extract lowered blood glucose levels by altering the activity of enzymes involved in glucose metabolism in the liver and by improving insulin resistance [141].

Dietary fibre present in seaweed such as alginates may reduce glycemic disturbances associated with obesity when included in the diet. A study involving forty self-reporting healthy males looked at the glycemic response to a controlled test lunch of varied composition following ingestion of an ionic-gelling alginate drink. It was seen that the alginate drink was able to attenuate the glycemic response following consumption of the test lunch [142].

One severe consequence of diabetes is the development of hyperglycemia-induced diabetic retinopathy (DR), a prevalent cause of blindness in many countries. LMWF from brown algae is known to demonstrate multiple biological activities (anti-inflammation, anti-oxidation, and anti-aggregation) which could be of benefit in treating ischemic disorders such as diabetic retinopathy. Calcium dobesilate is a strong antioxidant that is a current treatment for this condition. Mice with streptozotocin-induced diabetes were fed a diet containing LMWF (50, 100 or 200 mg/kg/day) or calcium dobesilate (50, 100 or 200 mg/kg/day) for four months to examine the protective role of the LMWF against the development of diabetic retinopathy, the production of high glucose-promoted vascular endothelial growth factor (VEGF) and the proliferation of cells in microvascular endothelial cells. The LMWF alleviated retinal pathological change and hindered neo-vascularization due to diabetes *in vitro* [143]

### 4.4. Obesity

Obesity is considered the gateway condition for several chronic diseases and is a major factor in the development of high blood pressure, type-2 diabetes, cardiovascular disease, and several types of cancer [144]. Obesity in children has been described as the most important health challenge of the 21st century, with the concern being that those individuals that are obese during their youth are likely to remain obese through to their adult life and as a result are more likely to develop cardiovascular diseases, cancer and diabetes [145]. One avenue of treatment is to manipulate the appetite and reduce the amount of food and calories consumed. A reduction in casual snacking between meals and in portion size would have a major impact on obesity levels [146]. Satiety is an important factor in regulating the amount of food that people consume and has a great importance in public health as a means of controlling obesity. Satiety or the feeling of fullness implies that there is a cessation of hunger as a consequence of consuming food. This is due to many factors including energy density, weight and volume, macronutrient composition, particle size, appearance of the food, satisfaction upon eating it and palatability [147]. Dietary alginates can slow down the rate that nutrients are absorbed into the gut and promote satiety both of which are of consequence in controlling obesity and type-2 diabetes. Following ingestion, alginate formulations react with gastric acid and undergo ionic gelation in the stomach to produce a gel that can reduce the rate of gastric emptying, stimulate gastric stretch receptors, reduce the uptake of nutrients and influence the glycaemic response after meals [148]. A human intervention study, investigating different alginate solutions intended for use as dietary supplements to enhance satiety and limit energy intake in humans, found that consumption of a formulation with a low ratio of mannuronic acid to guluronic acid resulted in a decrease in self-perceived capacity for food intake and increased sensation of fullness [149]. Researchers in Korea found that the intake of oily foods and seaweed were among the factors associated with a higher risk of developing metabolic syndrome. However, data from animal studies have suggested that seaweed intake may be protective against weight gain. Another study by Maeda and colleagues found that mice fed a diet containing fucoxanthin resulted in significantly reduced (*p* > 0.05) levels of abdominal white adipose tissue, blood glucose levels and insulin concentration [150]. Obesity can be characterised by an excessive deposition of fat with functional and morphological changes in adipocytes. The cell line 3T3-L1 is a preadipocyte cell line used in the study of adipogenesis. It has been shown that fucoxanthin enhances differentiation at an early stage but subsequently inhibits differentiation at the intermediate and late stages. Fucoxanthin also inhibited the uptake of glucose in mature 3T3-L1 adipocytes by reducing the phosphorylation of IRS-1 [130]. Fucoidan from the sporophyll of *U. pinnatifida* was investigated for anti-obesity potential through the inhibition of cytokines associated with inflammation. The presence of fucoidan significantly suppressed proliferator—activated receptor γ, C/EBPα, and adipocyte protein 2 while decreasing the expression of inflammatory-related genes in 3T3-L1 adipocytes during adipogenesis. Fucoidan also reduced the synthesis of ROS and the build-up of lipids in the cells [151]. *F. vesiculosus* has been claimed to be a useful agent for the management of obesity. *F. vesiculosus* contains large amounts of iodine which is believed to stimulate the thyroid gland and have a subsequent effect on the metabolic rate. The presence of high levels of dietary fibre, phytosterols and tetraterpenes are also important in helping obesity management [152].

## 5. Dietary Fibre, Seaweed Polysaccharides and Prebiotics

### 5.1. Dietary Fibre

**Figure 5:**
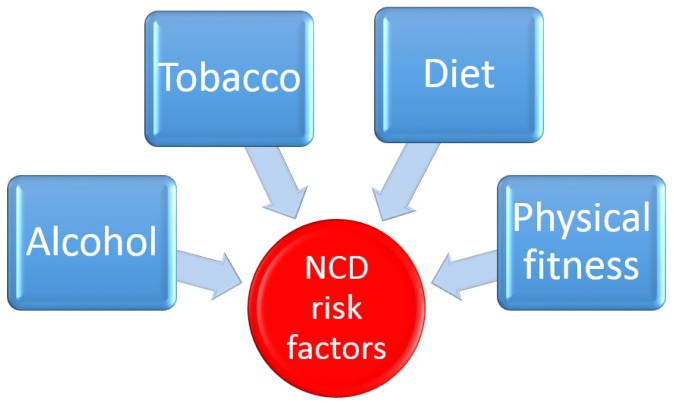
The major modifiable risk factors for the development of a chronic non-communicable disease are (1) Alcohol intake (2) Tobacco (3) Diet (4) Physical fitness

The main risk factors for NCDs for individuals are well known and are similar around the globe. Excess use of tobacco, harmful consumption of alcohol, low levels of physical activity and foods high in saturated trans fats, salts, and sugar account for two-thirds of all new cases of NCDs (Figure 5). In fact, the consumption of foods high in saturated and industrially produced trans fats, salt, and sugar is the cause of 40% of all deaths from NCDs [153]. A healthy diet coincides with lower incidence rates of CVD and other chronic diseases [154]. The dietary composition of humans has evolved greatly since the industrial revolution of the 18th and 19th centuries. Refined grains, meats, added fats and sugars have become more commonplace on the dinner table while the quantity of vegetables and fibre in our diets is reduced. This change in human nutrition, coupled with a more sedentary lifestyle is largely responsible for the increased level of obesity and other related chronic disease seen throughout the world today. Early humans had a predominantly plant based diet similar to what modern apes live on today. This diet was high in fibre and low in sugar and, based on current dietary guidelines, would be expected to impart low serum cholesterol levels. Due to our close genetic relationship with modern apes, it is thought that the drastic changes in dietary habits that have taken place in the last two hundred years may help to explain our present day problems with chronic illnesses such as type 2-diabetes, obesity and heart disease [155]. Seaweeds are a good source of minerals and nutrients that are important for many biochemical reactions. They are also rich in non-nutrient components such as dietary fibre and polyphenols [156]. Fibre is a generic term used to describe a broad family of carbohydrates found in plant cell walls. They are typically classified into three groups; soluble fibres (e.g., pectin and gums), insoluble fibres (e.g., cellulose) and mixed type fibre (e.g., brans). All fibre types share a common characteristic in that they are resistant to degradation by endogenous digestive tract enzymes, but can be broken down and fermented by the gut microbiota [157]. Dietary fibre is defined as the part of foodstuff that is not digested in the gastrointestinal tract (GIT). The chemical and physical properties of dietary fibre largely dictate its physiological effects. Soluble dietary fibres swell in the stomach and increase the density of the stomach content, hindering the absorption of nutrients in the intestinal mucosa. This effect can be beneficial in controlling non-insulin dependent diabetes as it causes a decrease in blood glucose and insulin responses after meals. Also an increased sense of satiety or fullness after eating may be useful in the treatment of obesity and the prevention of obesity-linked chronic diseases [158].

Seaweed polysaccharides are mainly found in their cell wall where they confer strength and flexibility to the plant as well as maintaining the cells internal ionic balance preventing desiccation [159]. The complexity of seaweed structural polysaccharides, such as agar (red seaweeds) and alginates (brown seaweeds) make them resistant to degradation by human digestive enzymes and therefore available for fermentation by the gut microbiota in the colon. As such they can be regarded as a source of dietary fibre. The dietary fibre content of seaweed can range from 33% to 75% with the soluble fraction consisting of as much as 50%–80% of total dietary fibre content [18]. In general, seaweed polysaccharides are hydrophilic, often water soluble and are known to establish intra-chain hydrogen bond networks making them rigid and stiff and ideal for use as thickeners. Seaweed polysaccharides also promote interactions with external ions and inter chain hydrogen bonding making them useful as gelling agents [159]. Dietary fibre obtained from seaweed differs in composition, chemical structure, physio-chemical properties and biological effects from terrestrially derived fibre sources [160]. In this regard, structural polysaccharides from brown seaweeds, such as laminarin and fucoidan could offer a dietary means to modulate the gut microbiota (as in the case of prebiotics—discussed below) and/or modulate immunity [161].

### 5.2. Prebiotics

There has been an increase in interest over the last two decades in the adjustment of the composition of the gut microbiota in order to confer a health benefit upon the host (human or animal). One particular area of research is of prebiotics. The prebiotic definition is constantly evolving as more information comes forth concerning the role of the gut microbiota in maintaining and promoting good health. The most recent definition of a dietary prebiotic is: “A selectively fermented ingredient that results in specific changes, in the composition and/or activity of the gastrointestinal microbiota, thus conferring benefit(s) upon host health” [162]. In order to be classified as prebiotic, several characteristics must first be met. The putative prebiotic must be able to resist digestion in the upper GIT, be selective in its stimulation of beneficial bacteria in the gut resulting in change in the profile of the microbiota and it must induce luminal or systemic effects that are beneficial to the health of the host [159]. Among the postulated health benefits of prebiotics for chronic conditions are: anti-colon cancer properties, osteoporosis management, improved bowel function, lipid lowering action, beneficial for cardiovascular disease associated with dyslipidemia and insulin resistance, obesity and possible type-2 diabetes. Seaweed derived polysaccharides (hydrocolloids) are potentially an important new source of prebiotics [163].

*Bifidobacterium* are well known for their ability to degrade complex carbohydrates in the colon and they are a common target for prebiotic ingredients. As a result, their genomes contain a relatively high number of genes (~8% of total genome) involved in the uptake of carbohydrates and metabolism, when compared to other commensal bacterial genomes [164]. For a recent review on carbohydrate metabolism of the *Bifidobacteria* see Pokusaeva *et al*. [165]. Prebiotics have been found to be of benefit in chronic inflammatory bowel disease in transgenic rats by preventing the development of colitis. The protective effect was seen in association with an increase in the number of intestinal *Bifidobacteria* and *Lactobacillus* [166]. An investigation by [167] looked at the effects of supplying diets containing seaweed derived laminarin and fucoidan to growing pigs. During their weaning phase, pigs are susceptible to carrying *Salmonella typhimurium* and other pathogens. It was found that such a supplemented diet resulted in an increase in *Lactobacillus* numbers in the caecum and an increase of butyric acid in the caecum and colon. Increased shedding of faecal *S. typhimurium* at selected time points during the experiment was also recorded. A recent study by Ramnani *et al.* [18], investigated the prebiotic and fermentation potential of low molecular weight polysaccharides (LMWP) derived from agar and alginate using pH and temperature controlled anaerobic batch cultures that were inoculated with human faecal matter. Fluorescent *in-situ* hybridization (FISH) was used to monitor changes in microbial composition and gas chromatography was utilised to monitor the fermentation end products, short chain fatty acids. It was found that the LWMP derived from *Gelidium* spp. showed a significant increase in bifidobacteria populations from log_10_ 8.06 at 0 h to log_10_ 8.55 at 24 h [18].

Alginates from seaweed have also been used as an encapsulation agent for the delivery of probiotics. Alginates are non-toxic, biocompatible, inexpensive to obtain and are easily solubilised in the human intestine facilitating the release of their entrapped cells [168]. Magnesium is in abundant supply in fibre rich foods such as seaweed. In animal trials, the intake of magnesium supplements prevented a drop in resistance to insulin or glucose intolerance and postponed the development of spontaneous diabetes mellitus. In humans, dietary fibre is believed to be inversely linked to the risk of developing diabetes mellitus. Results from human trials show that dietary fibre or fibre rich foods can improve the after meal glycemic response, most likely due to lower rates of glucose absorption and increased utilization of glucose in the gut. The effect of magnesium and fibre intake on the development of diabetes in 1604 healthy subjects aged above 30 was assessed [169]. A total of 141 diabetes incidents were recorded during the follow-up period and it was concluded from the dietary intake information that lower levels of magnesium, lower total dietary fibre intake, or a combination of both was associated with a higher risk of diabetes in the test population. The cell walls of some species of red seaweed contain the linear polymer agarose. The enzyme α-agarase can hydrolyse the α-1,3 linkage to produce agaro-oligosaccharide (AOS) while β-agarase works on the β-1,4 linkage yielding neoagaro-oligosaccharide (NAOS). NAOS was seen to be highly resistant to the enzymes of the upper GIT. NAOS significantly stimulated the growth of bifidobacteria and lactobacilli in MRS medium, compared with fructooligosaccharides (FOS), 1% (*w*/*v*) NAOS significantly promoted the specific growth rate of beneficial bacteria by approximately 100%. *In vivo*, NAOS significantly increased the numbers of lactobacilli and bifidobacteria (*p* < 0.05) in fresh feces or cecal content while reducing putrefactive microorganisms. NAOS with higher degrees of polymerization (DP) showed better prebiotic activity [170].

### 5.3. Production of Short Chain Fatty Acids (SCFA) by Colonic Bacteria and Health Benefits in Chronic Diseases

**Figure 6:**
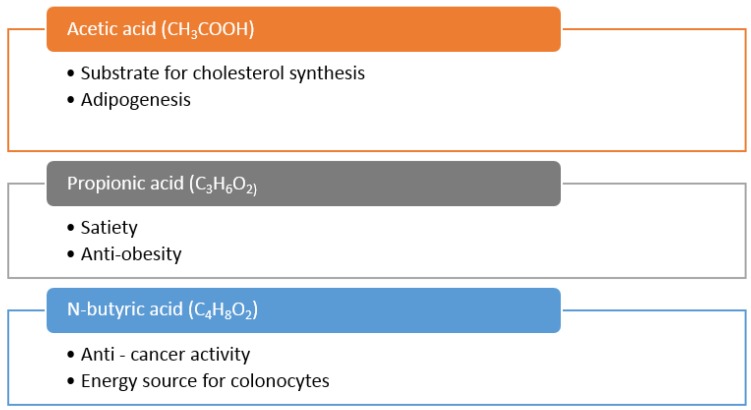
Putative health benefits of the three main short chain fatty acids (SCFAs), acetic acid, propionic acid and *N*-butyric acid. SCFA are mainly produced by the endogenous gut microbiota through the fermentation of undigested dietary fibres from the diet.

Metabolites of bacterial metabolism are affected by the different types of food that we eat and by the subsequent production of bacterial enzymes such as β-glucuronidase, β-glucosidase, mucinase and urease. Through their action, the intestinal lumen can be exposed to detrimental toxic, carcinogenic or mutagenic substances. By changing the substrates that are made available in the gut, and favouring the production of beneficial metabolites such as short chain fatty acids (SCFA), a healthier environment can be established [171]. For example, the anaerobic microbial communities that inhabit the mammalian gastrointestinal tract produce SCFA (acetic acid (acetate), butyric acid (butyrate) and propionic acid (propionate)) as their main non-gaseous dietary fibre fermentation end products [172]. SCFAs can impart several health benefits on the host and the intake of seaweed can alter the SCFA production profile of the microbiota (Figure 6). Propionate has been shown to: lower the fatty acid content in the liver and in plasma, reduce the amount of food eaten at meals, demonstrate immunosuppressive activity and help tissue sensitivity to insulin, all of which can be of benefit in the treatment and prevention of obesity and type-2 diabetes [173]. Butyric acid is a preferred substrate for colonocytes and appears to promote a normal phenotype in these cells [174] Butyrate has also received much attention as a chemoprotective agent for colorectal cancer [175] while acetate has been shown to increase colonic blood flow and enhance ileal motility [176].

When healthy Wister rats were fed the red seaweed *Mastocarpus stellatus* (Rhodophyta) (10% algal supplemented diet) an increase in the molar concentration of both acetate and propionate was seen while butyrate molar concentrations decreased. There was also a decrease in the total levels of SCFA produced between the algal treated group and the basal diet control group as well as an increase in caecal pH [177]. Work has been carried out to evaluate the effect of seaweed derived laminarin and fucoidan on different indices of GIT fermentation in newly weaned pigs. With regard to the production of the three main SCFAs, the addition of laminarin to pigs’ diet led to a significant increase in the concentration of acetic acid and a significant decrease in that of propionic acid in the caecum. The addition of fucoidan to their diet significantly increased acetic acid concentration and decreased the concentration of propionic acid in the same regions. Fucoidan also significantly increased butyric acid concentrations in both the caecum and the colon [167]. A study involving the *in vitro* fermentation of ten LMWP derived from agar and alginate from seaweed (*Gracilaria* spp. *Gelidium corneum* (formerly *G. sesquipedale*) (Rhodophyta) and *A. nodosum*) showed that the LMWPs caused a significant increase in total SCFA levels, especially acetic acid and propionic acid showing that they were readily fermented by the faecal bacteria [18].

### 5.4. Potential of Seaweed Components to Alleviate Respiratory Diseases and Allergies

Asthma is a complex inflammatory disease of the lungs characterised by variable airflow obstruction, airway hyper-responsiveness (AHR), and airway inflammation. The inflammatory response is characterised by infiltration of the airway wall by mast cells, lymphocytes and eosinophils and is associated with several inflammatory proteins, including cytokines, enzyme and adhesion molecules in the airway [178]. It is estimated that some 235 million people currently suffer from asthma but it is likely that asthma is both under-diagnosed and undertreated worldwide. Asthma is the most common chronic disease affecting children. The fundamental cause of asthma is not completely known, but the use of medication and the avoidance of certain environments and triggers can reduce the severity of the condition. Triggers include indoor allergens such as dust mites, and pet dander; outdoor allergens like pollen and moulds; tobacco smoke; chemical irritants in the workplace and air pollution. Exposure to cold air and extreme emotional arousal (fear, anger) can also bring about attacks [179].

Polyphenolic extracts from the edible *Chondrophycus undulatus* (formerly known as *Laurencia undulata*) (Rhodophyta) have been shown to possess therapeutic potential for combating broncial asthma associated with allergic diseases. Mice sensitised and challenged with ovalbumin (OVA) showed typical asthma symptoms as follows: an increase in the number of eosinophils in the bronchoalveolar lavage (BAL) fluid; a marked influx of inflammatory cells into the lung around blood vessels and airways, and airway luminal narrowing; airway hyper-responsiveness; detection of TNF-α and TH2 cytokines in the BAL fluid; and detection of allergen specific IgE in the serum. Intraperitoneal treatment of *L. undulata* extracts before the last airway OVA challenge resulted in significant inhibition of all asthmatic reactions [178]. Previously, extracts from the *E. cava* were also seen to be effective in relieving asthma symptoms in sensitised mice challenged with OVA by inhibiting the Th2 response [180].

Allergic diseases affect approximately one third of the general population. They are caused by chemical or immunological activation of mast cells leading to massive release of endogenous mediators such as histamine and a wide variety of inflammatory mediators such as eicosanoids, proteoglycans, proteases and several pro-inflammatory and chemotactic cytokines such as TNF-α, interleukin (IL-6, IL-4, IL-13) Preventing immune cells from degranulating is one of the crucial steps in preventing an allergic disorder. Two phlorotannins isolated from *E. cava*, 6,6′-bieckol and 1-(3′,5′-dihydroxyphenoxy)-7-(2″,4″,6-trihydroxyphenoxy)-2,4,9-trihydroxydibenzo-1, 4-dioxin were found to exhibit anti-allergic activities. The proposed mechanism of activity was that the seaweed compounds prevented degranulation by suppressing the binding of IgE and the FcεR receptor [181].

Allergic rhinitis (hay fever) is an inflammatory nasal disorder that involves activation and tissue recruitment of both structural cells and infiltrating leukocytes [182]. In a study to investigate a possible protective effect of the traditional Japanese diet on allergic disorders, it was observed that a high dietary intake of seaweed may be associated with a decreased prevalence of allergic rhinitis (hay fever) [183]. A high serum IgE concentration is a defining characteristic of atopic diseases such as atopic asthma and allergic rhinitis, with levels correlating with the extent and severity of the disease. Fucoidan from seaweed has been shown to reduce the increase of IgE in mice exposed to OVA. Fucoidan inhibited IgE production by preventing the NFκB p52-mediated pathways activated by CD40 [184].

## 6. Conclusions

Dealing with a chronic illness on a day to day basis is a fact of life for an ever increasing proportion of the world’s population. This is an unfortunate knock-on effect of extended human longevity and a decrease in incidence rates of infectious disease. The most important preventive measure one can take to avoid developing a chronic, non-communicable disease is to modify diet and lifestyle factors. It is well documented that excessive use of tobacco and alcoholic beverages, not being physically active or eating unhealthily will greatly increases an individual’s chances of developing an NCD. However, for millions of people already suffering from chronic illness, simply avoiding risk factors is no longer an option. As the incidences of NCDs rise in the years to come, the burden on the world’s heath care services will also increase. New therapies will be sought out to provide better care and more economical services for long term patients. The emphasis up until now has been in searching the terrestrial regions of the planet for new drugs and bioactives. However, to face the new challenges of the future, new environments must be explored. The surface of the Earth is over 70% water and within the marine environments (the oceans, seas, rivers and lakes) of the planet, there exists an immense quantity of biological diversity with untapped potential. Seaweeds are a common sight along the coastal regions of the world. These marine plants have a long tradition of diverse use by mankind having being used for centuries in food preparation and traditional medicine. It is only in relatively recent times that the scientific community has developed the capabilities to better understand the health benefits of seaweeds that our ancestors knew of anecdotally. Indeed, the literature presented here clearly demonstrates the plethora of novel bioactives that seaweed has to offer. While much of the research heralds the therapeutic effects from *in vitro* studies, the way has been laid to assess their efficacy *in vivo* through extensive animal trials and human clinical studies.

In conclusion, there is little doubt that the fight to control the rise of chronic, NCDs will be a major challenge in the 21st century, but it is not an insurmountable one. The further education of current and future generations in making positive healthy lifestyle choices along with increased scientific research into the underlying causes of chronic disease and the development of new treatments can serve as a platform for effective future therapies.

## Figures and Tables

**Table 1:** A wide variety of biological activities have been reported from algal polysaccharides. This table outlines observed activities in some recent studies.

Algal Polysaccharide	Extraction Method	Seaweed	Reported Activity	Reference
Fucoidan	Hot water extraction	*Sargassum glaucescens* (Phaeophyceae)	Anti-oxidant activity	[48]
Fucoidan	n/a	*Sargassum fusiforme* (Phaeophyceae)	Cognitive protective activity	[49]
Fucoidan	Ethanol extraction	*Isostichopus badionotus* (Stichopodidae)	Anti-inflammatory activity	[50]
Fucoidan	Ethanol extraction	*S. fusiforme*	Anti-angiogenic activity	[51]
Fucoidan	Ethanol extraction	*Coccophora langsdorfii* (Phaeophyceae)	Anti-cancer activity	[52]
Fucoidan	Methanol extraction	*Sargassum swartzii* (Phaeophyceae)	Anti-viral activity	[53]
Fucoidan	Ethanol extraction	*Fucus vesiculosus* (Phaeophyceae)	Anti-hyperglycemic activity	[54]
Fucoidan	n/a	*F. vesiculosus*, *Ascophyllum nodosum* (Phaeophyceae)	Anti-diabetic activity	[31]
Laminarin	n/a	n/a	Anti-fungal activity	[55]
Laminarin, Fucoidan	n/a	*Laminaria digitata* (Phaeophyceae)	Anti-oxidant activity	[56]
Agar, alginates	n/a	*Gelidium* sp., *Gracilaria* sp., and *A. nodosum*	Prebiotic activity	[18]
Alginic acid	n/a	n/a	Anti-oxidant activity	[57]
Alginate	n/a	*Durvillaea* sp., *Lessonia nigrescens* (Phaeophyceae)	Anti-obesity activity	[58]
